# Setting the international research agenda for sarcomas with patients and carers: results of phase II of the Sarcoma Patient Advocacy Global Network (SPAGN) priority setting partnership

**DOI:** 10.1186/s12885-024-12732-6

**Published:** 2024-08-06

**Authors:** E. Roets, K. Schuster, S. Bickley, M. Wartenberg, O. Gonzato, N. Fernandez, B. Kasper, K. Pilgermann, R. Wilson, N. Steeghs, W. T. A. van der Graaf, G. van Oortmerssen, O. Husson

**Affiliations:** 1 Medical Oncology Department, Netherlands Cancer Institute, Plesmanlaan 121, 1066 CX, Amsterdam, The Netherlands; 2 Sarcoma Patient Advocacy Global Network (SPAGN), Untergasse 36, D-61200 Wölfersheim, Germany; 3 Policy and Support, Sarcoma UK, 17/18 Angel Gate City Road, London, UK; 4 German Sarcoma Foundation, National Center for Tumor Diseases, Heidelberg, Germany; 5 Fondazione Paola Gonzato-Rete Sarcoma ETS, Rome, Italy; 6 Sarcoma Unit, Mannheim University Medical Center, Heidelberg, Germany; 7 Sarcoma UK, 17/18 Angel Gate City Road, London, UK; 8 Medical Oncology Department, Erasmus Cancer Institute, Erasmus University Medical Center, Doctor Molewaterplein 40, 3015 GD, Rotterdam, The Netherlands; 9 Surgical Oncology Department, Erasmus Cancer Institute, Erasmus University Medical Center, Doctor Molewaterplein 40, 3015 GD, Rotterdam, The Netherlands

**Keywords:** Sarcoma, Gastrointestinal stromal tumor, Desmoid fibromatosis, Bone sarcoma, Soft tissue sarcoma, Priority setting partnership, Patient involvement, Patient advocacy

## Abstract

**Background:**

Typically, researchers and clinicians determine the agenda in sarcoma research. However, patient involvement can have a meaningful impact on research. Therefore, the Patient-Powered Research Network (PPRN) of the Sarcoma Patient Advocacy Global Network (SPAGN) set up a Priority Setting Partnership (PSP). The primary objective of this partnership is to identify priorities for research and patient advocacy topics.

**Methods:**

In the first phase of this PSP, including 264 sarcoma patients and carers from all over the world, 23 research topics regarding sarcomas and 15 patient advocacy topics were identified using an online survey. In the second phase, participants were asked to fill in a top five and a top three of research and patient advocacy topics, respectively. Additionally, sociodemographic characteristics and sarcoma characteristics were collected. Social media channels, local national patient advocacy groups and the SPAGN website were used to distribute the survey.

**Results:**

In total, 671 patients (75%) and carers (25%) participated in this survey. The five highest ranked research topics were related to causes of sarcoma (43%), prognosis and risk of recurrence (40%), specific subtypes of sarcoma (33%), the role of immunotherapy, targeted therapy and combined therapy (30%), and hereditary aspects (30%). The three highest ranked patient advocacy topics were improving the diagnostic process of sarcoma (39%), access to tumor DNA analysis (37%) and establishing an international sarcoma registry (37%).

**Conclusions:**

This sarcoma PSP has identified priorities for research and patient advocacy, offering guidance for researchers, assisting funding agencies with assessing project relevance and empowering patient advocates to represent the needs of patients and carers.

**Supplementary Information:**

The online version contains supplementary material available at 10.1186/s12885-024-12732-6.

## Introduction

Sarcomas are a heterogeneous group of tumors, originating from mesenchymal cells, with an incidence in Europe of 6.1 per 100 000 persons per year and therefore account for less than 1% of all solid malignant cancers [1, 2]. A broad distinction can be made between bone sarcomas (BS) and soft tissue sarcomas (STS). Today, the World Health Organisation classification includes approximately 100 different sarcomas [3]. Tumors related to STS but often considered separately in the context of research, are gastrointestinal stromal tumors (GISTs) and desmoid fibromatosis (DF). GIST is the most common gastrointestinal sarcoma subtype [4, 5] and DF is a borderline mesenchymal tumor characterized by infiltrative growth but an inability to metastasize [6]. The clinical behaviour and treatment vary according to the histological subtype, leading to many challenges for sarcoma patients, clinicians, researchers and caregivers.

The traditional approach, where researchers and healthcare professionals (HCPs) determine the agenda in sarcoma research is changing [7–12]. This is nicely reflected by the creation of patient-centered organisations and patient advocacy groups that pursue to integrate the patient’s voice into the process of prioritizing research needs. Several studies demonstrated that patient involvement can have a meaningful impact on research [13–17]. Despite these efforts, there is a mismatch in research needs felt by patients on one hand and health professionals on the other hand [18, 19]. This was the trigger to create the James Lind Alliance (JLA), which brings together patients, carers and clinicians in Priority Setting Partnerships (PSP) in order to identify and prioritise uncertainties in specific areas in the medical field that could be answered by research [20]. While multiple JLA-based PSPs have successfully identified research questions, this hasn’t been the case for sarcomas [7–11]. Therefore, the Patient-Powered Research Network (PPRN) of SPAGN (Sarcoma Patient Advocacy Global Network), a global network of national sarcoma patient advocacy organisations, set up a Priority Setting Partnership (PSP), based on the JLA methodology.

In the first phase of this PSP, research topics regarding sarcomas and topics for patient advocacy groups were identified using an online questionnaire [12]. This resulted in 23 research topics and 15 patient advocacy topics, identified by 264 respondents. This study reports on the second phase of the PSP and aims to prioritize the research and patient advocacy topics identified in the first phase of the survey and to investigate differences in prioritization between subgroups of respondents [12].

## Methods

This PSP was set up by SPAGN in collaboration with other stakeholders in the sarcoma research field, according to the JLA methodology. This methodology involves engaging patients, carers, and HCPs in PSPs to collaboratively identify and prioritize research questions, and is extensively described in the previous publication [12].

### Questionnaire and respondents

Research topics and patient advocacy topics identified during the first phase of this PSP were rephrased to 24 research topics and 14 patient advocacy topics, respectively. Research topics focused on the origin of sarcomas, the diagnostic process, treatment and side effects, prognosis, quality of life (QoL) and end of life. Topics for patient advocacy focused on the diagnostic process, communication between patients and HCPs, data sharing (i.e. sharing of relevant patient data across medical centers), information on tumor subtypes, QoL, end of life, expert centers and off-label or compassionate use medications.

Eligible respondents included patients and survivors of sarcoma (including GIST and DF), carers or family members of people who have (had) sarcoma and patient advocates. The questionnaire was specifically developed for this study and consisted of three sections (supplementary 1). In sections one and two, respondents were asked to fill in a top five and a top three of research and patient advocacy topics, respectively. One open question was included where patients could address any missing research topics not listed in the survey. Section three assessed, the respondents’ connection with sarcomas, sociodemographic characteristics (i.e. age, gender, ethnic origin, educational level, comorbidities, residence country) and sarcoma characteristics (i.e. tumor type and location, disease stage, treatment intention, treatment type).

In addition to English, the survey was translated to Dutch, Bulgarian, Finnish, French, German, Hindi, Italian, Japanese, Polish, Swedish and Spanish, to assure a demographic variety of respondents. The questionnaire was distributed via the website of SPAGN, social media channels and via local national patient advocacy groups. The questionnaire was posted online using the LimeSurvey platform and was open for completion from April 2023 until July 2023 [21].

### Analysis

The analysis was conducted using Excel. Results were summarized in table format, reporting absolute numbers and frequencies. For each question the number of missing data was specified. Priorities for research and patient advocacy topics were described for different subgroups, including tumor type (i.e. BS, STS, GIST and DF), the top five respondent countries, gender, age groups (adolescents and young adults (AYAs) versus older adults), patients (including patient advocates) and carers, indication of treatment (i.e. curative and palliative). AYAs were defined as patients aged between 15 and 39 years.

## Results

### Respondents’ sociodemographic characteristics

The survey was completed by 671 individuals. Sociodemographic characteristics are listed in Table 1. Most respondents were female (82%) and the median age was 50 years (range 19–80). The majority of respondents were sarcoma patients or patient advocates (75%), followed by (bereaved) carers/relatives (25%). Most of the respondents described their situation as under regular follow-up (39%), part-way through their treatment (26%) or finished with treatment (13%). The majority of respondents had a college diploma (30%) or a university degree (44%). Most of the respondents were living in Germany (19%), followed by the Netherlands (14%), Japan (13%), the United Kingdom (12%) and Italy (11%).

**Table 1:** Sociodemographic characteristics

	*N* = 671 (%)
**Gender**	
Male	112 (17)
Female	549 (82)
Missing	10 (1)
**Age (years)**	
Median (range)	50 (19–80)
Prefer not to say	15 (2)
Missing	5 (1)
**Respondents’ connection with sarcoma** ^**a**^	
Sarcoma patients and patient advocates	501 (75)
(Bereaved) Carer/partner/relative of sarcoma patient	170 (25)
**Comorbidities**	
Yes	189 (28)
No	433 (64)
‘I don’t know’	23 (3)
Missing	26 (4)
**Ethnic origin**	
White	517 (77)
African American/black	2 (< 1)
Asian or Pacific Islander	115 (17)
Hispanic/Latino	9 (1)
Multiple/mixed	8 (1)
Other^b^	8 (1)
Missing	12 (2)
**Educational level**	
No education/primary school	2 (< 1)
Secondary school	155 (23)
College/diploma	203 (30)
University/degree	295 (44)
Other^c^	1 (< 1)
Missing	15 (2)
**Country of residence**	
Japan	90 (13)
Netherlands	94 (14)
Germany	127 (19)
United Kingdom	80 (12)
Spain	11 (2)
Italy	74 (11)
United States of America	37 (6)
Belgium	29 (4)
Finland	22 (3)
France	17 (3)
India	21 (3)
Other^d^	42 (6)
Prefer not to say	13 (2)
Missing	14 (2)

^a^ Some patients had multiple answers (e.g. ‘sarcoma patient’ and ‘patient advocate’)

^b^ Turkish

^c^ Not specified

^d^ Australia (*n* = 2), Austria (*n* = 4), Brazil (*n* = 4), Bulgaria (*n* = 6), Canada (*n* = 7), Czech Republic (*n* = 1), Denmark (*n* = 1), Indonesia (*n* = 1), Iraq (*n* = 1), Luxembourg (*n* = 2), Macedonia (*n* = 1), New Zealand (*n* = 2), Poland (*n* = 3), Portugal (*n* = 1), Switzerland (*n* = 1), Sweden (*n* = 4), Vietnam (*n* = 1)

### Sarcoma characteristics

Sarcoma characteristics are presented in Table 2. The majority of patients had a STS (53%) with liposarcoma (11%) and leiomyosarcoma (10%) as most common STS subtypes. In most cases the sarcoma was located intra-abdominal (24%), in the lower limb (23%), upper limb (11%), retroperitoneal (10%) or torso (12%). Patients had localized (66%) or metastatic disease (25%) and were receiving/had received curative (52%), palliative therapy (22%) or best supportive care (7%). The disease stage and the intention of treatment were missing or stated as ‘unknown’ by the patient, in 9% and in 18% of cases, respectively. Most patients were diagnosed with a sarcoma within the past one to five years (42%).

**Table 2:** Characteristics of the sarcoma (*n* = 671)

	*N* = 671 (%)
**Tumor type**	
Bone sarcoma	83 (12)
Soft-tissue sarcoma	354 (53)
Gastrointestinal stromal tumour	112 (17)
Desmoid fibromatosis	94 (14)
Phyllodes	15 (2)
‘I don’t know’	3 (< 1)
Other^a^	5 (1)
Missing	5 (1)
**Subtype of soft tissue sarcoma or bone sarcoma**	
Angiosarcoma	21 (3)
Chondrosarcoma	40 (6)
Leiomyosarcoma	67 (10)
Liposarcoma	72 (11)
Synovial sarcoma	26 (4)
Ewing sarcoma	31 (5)
DFSP	13 (2)
Myxofibrosarcoma	17 (3)
Sarcoma NOS/undifferentiated sarcoma	26 (4)
Osteosarcoma	31 (5)
Rhabdomyosarcoma	11 (2)
Spindle cell sarcoma	7 (1)
Epitheloid sarcoma	6 (1)
Endometrial stromal sarcoma	8 (1)
Epitheloid hemangioendothelioma	5 (1)
Desmoplastic small round cell tumor	5 (1)
Alveolar soft part sarcoma	5 (1)
‘I don’t know’	11 (2)
Other	31 (5)
Missing	4 (1)
**Sarcoma location**	
Upper limb	71 (11)
Lower limb	156 (23)
Head/Neck	30 (4)
Retroperitoneal	67 (10)
Heart/vascular	10 (1)
Gynaecologic	58 (9)
Intra-abdominal	158 (24)
Torso	82 (12)
Multiple locations	10 (1)
Other^b^	27 (4)
Missing	2 (< 1)
**Disease stage**	
Localized	441 (66)
Metastatic	171 (25)
‘I don’t know’	32 (5)
Missing	27 (4)
**Intention of treatment**	
Curative	350 (52)
Palliative	149 (22)
Best supportive care	47 (7)
‘I don’t know’	55 (8)
Missing	70 (10)
**Treatments received/receiving**	
Surgery	519 (77)
Amputation	44 (7)
Radiotherapy	208 (31)
Chemotherapy	275 (41)
Proton beam therapy	18 (3)
Targeted therapy	104 (15)
Immunotherapy	31 (5)
Hormonal therapy	38 (6)
Isolated limb perfusion	7 (1)
Hyperthermia	14 (2)
I took/am taking part in a clinical trial	41 (6)
I am not treated (e.g. watchful waiting)	28 (4)
‘I don’t know’	3 (< 1)
Other^c^	13 (2)
**Number of years living with sarcoma**	
< 1 year	153 (23)
≥ 1 year - < 5 years	281 (42)
≥ 5 years - < 10 years	108 (16)
≥ 10 years	98 (15)
Missing	31 (5)

^a^ Myeloid sarcoma (*n* = 1), giant cell tumor (*n* = 4)

^b^ Abdominal, not specified (*n* = 4), abdominal wall (*n* = 6), extra-abdominal (*n* = 1), groin (*n* = 1), lung (*n* = 5), mediastinum (*n* = 2), not applicable (patient advocate; *n* = 1), fossa ischiorectalis (*n* = 1), scalp (*n* = 4), spermatic cord (*n* = 2)

^c^ Alternative therapies (*n* = 2), ablation (*n* = 7), HIFU (*n* = 1), embolization (*n* = 1), anti-inflammatory drugs (*n* = 2)

### Research priorities

#### Research priorities in the overall group

The ranking of research topics for the entire group of respondents is listed in Table 3. The five highest ranked research topics were related to causes of sarcomas (43%), prognosis and risk of recurrence (40%), specific subtypes of sarcomas (33%), the role of immunotherapy, targeted therapy and combined therapy (30%), and hereditary aspects (30%).

**Table 3:** Top 5 of unanswered research questions and topics

	*N* (%)
1. What are causes of sarcoma?	288 (43)
2. What is the prognosis and the risk of recurrence of sarcoma and which factors have an effect on this?	269 (40)
3. More research specific on subtypes of sarcoma (e.g. GIST, retroperitoneal liposarcoma, angiosarcoma, …) is needed.	219 (33)
4. What is the role of immunotherapy, targeted therapy and combined therapy in the treatment of sarcomas?	199 (30)
5. In which way are hereditary aspects involved in the development of sarcoma?	198 (30)
6. Are there ways to prevent sarcoma?	191 (28)
7. What is the effect of different treatment modalities on survival and quality of life?	184 (27)
8. What are the most accurate techniques for the diagnosis of sarcoma (think of imaging modalities, blood tests, whole genome sequencing, etc.) and which techniques or strategies could be used to improve the distinction between different subtypes of sarcoma and between benign and malignant tumors?	183 (27)
9.What is the effect of lifestyle (diet, physical activity, etc.) on the development of sarcoma?	166 (25)
10.Can vaccines be developed to prevent or treat sarcomas?	146 (22)
11. What is the effect of different surgical techniques and surgical margins on the outcome for the patient (think of functional outcomes, prognosis, recurrence, etc.)?	144 (21)
12. Which personal characteristics have sarcoma survivors in common (think of psychological, medical and sociodemographic characteristics)?	137 (20)
13. What are the side effects of the different treatment options (targeted therapy, chemotherapy, radiotherapy, surgery, etc.) and how can these side effects be treated?	113 (17)
14. What is the effect of lifestyle on the outcome (e.g. quality of life) during and after treatment?	101 (15)
15. What percentage of people with sarcoma receive the wrong diagnosis in the first instance?	94 (14)
16. What is the risk of taking a biopsy?	93 (14)
17. How can follow-up scheme for sarcoma patients be better personalised?	92 (14)
18. What is happening in the terminal phase (development of the disease) and what are the best methods to give best supportive care?	86 (13)
19. What are the possible treatment methods (e.g. psychotherapy, mindfulness, psychedelics) for disease-related mental suffering (e.g. acceptance, anxiety)?	75 (11)
20. More research is needed into novel surgery techniques.	73 (11)
21. What are the long-term effects of sarcoma treatment on intimacy and fertility?	39 (6)
22. How can the re-integration of sarcoma survivors in the society be facilitated (think of work re-integration, social re-integration)?	32 (5)
23. What is the role of carers in the final phase of life and how can carers support the patient in taking decisions in the final phase of life?	28 (4)
24.How is end-of-life care organised (in different countries)?	26 (4)

#### Research priorities in respondent subgroups

In the subgroup of GIST patients and carers, 55% prioritized research on specific subtypes of sarcoma, whereas for the subgroups of BS, STS and DF this was 35%, 31% and 12%, respectively (supplementary 2). Among DF patients and carers, 50% prioritized research into the effect of lifestyle on the development of the tumor, whereas for the subgroups of BS, STS and GIST this was 20%, 19% and 24%, respectively. In the subgroup of AYA patients (*n* = 105) research topics addressing the effect of lifestyle on the development of sarcomas and the long-term effects of sarcoma treatment on intimacy and fertility were prioritized more often compared to older adults (*n* = 391) (34% and 22% versus 24% and 2%, resp.) (supplementary 3). Broadly, the top five of research priorities for the overall group matched those of the top 5 respondent countries. However, in the subgroup of Italian respondents, the research topic addressing the effect of lifestyle on the development of sarcomas featured among the top five (38%), whereas this was lower ranked in other countries (range 13–30%) (supplementary 4). Japanese respondents highly prioritized research focusing on specific sarcoma subtypes (56%), while this was prioritized by only 15% of Italian respondents. No clear differences were observed in research priorities between genders (supplementary Table 5). 20% of carers prioritized research regarding the terminal phase of the disease (i.e. development of the disease and methods to give best supportive care), whereas this was prioritized by only 10% of sarcoma patients (supplementary Table 6). Among patients treated in a palliative setting, 31% prioritized research regarding the terminal phase of the disease and 38% prioritized research about the effect of different treatment modalities on survival and QoL (supplementary Table 7). In contrast, these aspects were prioritized by 7% and 21%, respectively, of patients treated in a curative setting.

### Patient advocacy priorities

#### Patient advocacy priorities in the overall group

The ranking of patient advocacy topics for the entire group of respondents is listed in Table 4. The three highest ranked patient advocacy topics were improving the diagnostic process of sarcomas (39%), access to tumor DNA analysis (37%) and establishing an international sarcoma registry (37%).

**Table 4:** Top 3 of patient advocacy topics

	*N* (%)
1. Improving the diagnostic process of sarcoma through better education and development of tools that can assist general practitioners in recognizing the possibility of a sarcoma.	264 (39)
2. Analysis of the tumor DNA should be available for all patients.	249 (37)
3. An international registry with data about sarcoma patients is needed to supply data for research and stimulate international research collaboration.	246 (37)
4. Referral of patients to sarcoma expert centers, centralization, networks.	163 (24)
5. Data sharing should be improved; all relevant data of a patient should be available across medical centers.	151 (23)
6. The availability to patients of off-label or compassionate use medication.	149 (22)
7. More attention should be given to quality of life and consequences of treatment (e.g. pain, temporary/permanent effects of surgery, side effects of medication) during the shared-decision making process.	147 (22)
8. Sarcoma centers should advise patients on complementary treatments, lifestyle and diet.	140 (21)
9. Communication between specialists and patient must be improved to stimulate shared decision-making.	126 (19)
10. Mental support must be available for sarcoma patients.	114 (17)
11. A single point of contact must be provided to patients (e.g. case manager, specialized nurse).	93 (14)
12. A better classification is needed for benign and malignant tumors. Benign tumors should be included in tumor registries.	92 (14)
13. Information on all tumor subtypes must be available for patients.	44 (7)
14. End-of-life scenario should be discussed openly and timely with the patient.	41 (6)

#### Patient advocacy priorities in subgroups

Among desmoid patients and carers the patient advocacy topic ‘classification of benign and malignant tumors’ featured among the top 5 (40%), while this was prioritized less frequently by respondents of other tumor groups (range 6–24%) (supplementary Table 8). Improvement of the diagnostic process was ranked highest in the subgroup of BS patients and carers (60%). In the subgroup of AYA patients mental support was prioritized higher compared to the subgroup of older adults (30% versus 15%) (supplementary Table 9). Broadly, patient advocacy priorities identified in the overall group of respondents aligned well with the priorities across countries (supplementary Table 10). 34% of Dutch patients prioritized the topic addressing ‘attention to QoL and consequences of treatment during the shared-decision making process’ (range in other countries: 13–23%). No clear differences were observed in patient advocacy priorities between males and females (supplementary Table 11), between patients and carers (supplementary Table 12) and curative versus palliative treatment indications (supplementary Table 13).

## Discussion

This is the first PSP bringing together sarcoma patients, carers, researchers and HCPs. The top five of research topics and the top three of patient advocacy topics, identified by this study, could provide guidance for researchers, policy-makers, caregivers and patient advocates. Interestingly, priorities differed across specific subgroups (e.g. tumor subgroups, age groups, etc.).

The research priorities identified in this survey reflect the characteristics of sarcoma etiology, diagnosis, treatment and prognosis. In contrast to other cancer types, for sarcomas very few risk factors are known [22]. This could explain why research questions related to causes of sarcomas and hereditary aspects were ranked high. In particular, research regarding the effect of lifestyle (e.g. diet, physical activity) on the development of sarcomas was highly ranked in the DF subgroup, possibly due to the scarcity of identified risk factors for sporadic desmoid tumors [23]. Also in the subgroup of Italian respondents this research topic was highly ranked, which might be explained by cultural factors (e.g. more public attention to the relation between lifestyle and onset of diseases in general). Additionally, this PSP highlighted the need for research focusing on the diagnostic process, given the frequent delay in sarcoma diagnosis [24]. One way to improve the diagnostic process could be to use real-world data from general practitioners to gain more insight into symptom patterns specific for sarcoma patients [25]. In contrast to other cancer types, the progress in improving survival of sarcoma patients has been limited, with a median OS in advanced STS patients of only 12 months [26, 27] and despite great efforts in sarcoma research, the past 10 years, there only has been a limited number of positive clinical trials and few new therapeutic options for STS patients [28–37]. While some RCTs have shown that there might be a role for immunotherapy in the treatment of sarcomas, better patient selection is needed to identify those who could benefit from treatment with immunotherapy [38]. Therefore it is not surprising that the development of novel drug therapies, including targeted therapy, immunotherapy and combined therapy was prioritized high [39]. In addition, the prognosis of sarcoma patients is highly variable and multiple tools to predict recurrence and OS have been developed, including the Sarculator and Personalised Sarcoma Care (PERSARC) [40, 41]. These tools can be used to assess the indication for (neo)adjuvant treatment, improve patient-tailored management and support the decision-making process. However, these tools are only applicable for localized (extremity) STS and to improve the accuracy addition of other predictors is required (e.g. gene expression profiles). This explains the high ranking of research focusing on recurrence and prognosis.

In contrast to JLA-based PSPs conducted in other cancer types, some research topics were ranked surprisingly low [11, 42–44]. For example, research topics addressing support during the final phase of life and organization of the end-of-life were prioritized by only 4% of respondents. This might be explained by the fact that the majority of patients was diagnosed with localized disease. Furthermore, results might be biased as patients in more critical health conditions might not have had the opportunity to participate in the survey. Moreover, given that 75% of respondents are patients, this topic might have been prioritized low due to the use of avoidance coping strategies driven by fear of death [45].

In terms of patient advocacy, creating international registries containing data from sarcoma patients was prioritized by 37% of respondents. Establishing international registries is vital in overcoming challenges when researching rare cancer types, such as sarcomas. Registries could contribute to sarcoma research by enabling larger sample sizes, facilitating comprehensive analyses, and improving the understanding and management of sarcomas. This aligns well with the concept of the Retroperitoneal Sarcoma Registry (RESAR), initiated by the TransAtlantic Retroperitoneal Sarcoma Working Group (TARPS), which aims to prospectively collect data from primary retroperitoneal sarcoma patients in Europe and North America [46]. 70% of respondents were residents of Germany, Netherlands, Japan, UK or Italy and only 7% of respondents prioritized information provision about tumor subtypes as a patient advocacy topic. This observation could imply that those countries provide sufficient information concerning tumor subtypes. Moreover these results indicate that most respondents likely have a connection with a patient advocacy group as they participated in the survey, which was primarily distributed by patient advocacy groups.

This study also highlighted distinct priorities among AYA patients, who prioritized mental support more often (30%) compared to older adults (15%). AYAs represent a vulnerable group of sarcoma patients as they have a different spectrum of cancer types compared to older adults and they are confronted with cancer during the most challenging time in their lives, leading to disruptions in their everyday life, social and professional life. Similar results were shown in a UK survey where mental support for AYAs with cancer belonged to the top 3 of research priorities [10]. AYAs are in the phase of their life exploring their sexuality and body-image and both the sarcoma diagnosis and the treatment itself can interfere with their sexual development. While a survey among AYAs with cancer in the Netherlands showed that communication about sexuality was considered crucial, the majority of AYAs was not satisfied with the provided information [47]. This unmet need was also identified in our survey with 22% of AYAs prioritizing research about long-term effects of sarcoma treatment on intimacy and fertility (versus 2% in older adults). Reintegration into daily life after surviving cancers might be very challenging for AYAs. Nevertheless, only 6% of AYAs prioritized the research topic focusing on the re-integration of sarcoma survivors into society. This doesn’t necessarily imply that this topic lacks relevance, but rather suggests that other topics were given higher priority.

One of the strengths of this study is that priorities for research and patient advocacy were assessed in various subgroups, allowing to gain more insight in the needs of various respondent subgroups and facilitating a more nuanced interpretation of results. Furthermore, we reached a high number of respondents and with more than 30 tumor types, respondents displayed a wide range of tumor types.

This sarcoma PSP has several limitations. The questionnaire was translated into 11 languages and respondents of more than 20 different countries were involved. Nevertheless, with the majority of respondents being higher educated and predominantly female respondents, these results are not representative for the entire sarcoma community. This problem is often encountered in survey research and has been observed in other PSPs as well [48, 49]. Future sarcoma PSPs should aim to reach the underrepresented groups by using clear and understandable language and by offering different modalities for survey participation (e.g. online, paper). Another limitation might be the lack of specific research questions that are only relevant to a specific subgroup of respondents, potentially resulting from elimination of these questions during the first phase of the PSP. Another limitation is that topics were not presented in a random order each time the survey was opened. This might induce bias with topics at the bottom of the list being less frequent prioritized.

The PSP methodology is being applied worldwide for different disease entities and has directly lead to the initiation of multiple research projects [50, 51]. This sarcoma PSP has identified priorities for research and patient advocacy, offering guidance for researchers, assisting funding agencies with assessing project relevance and empowering patient advocates to represent the needs of patients and carers. In order to successfully influence sarcoma research and patient advocacy multiple post-PSP processes are needed, including propagation of the top priorities (e.g. using networks of patient organisations, scientific conferences, etc.), involve funders, translation of research priorities into research projects, keep track of priorities that have been assessed and share details of progress with different stakeholders (e.g. patients, HCPs, carers, etc.). Moreover, this study showed that telemedicine and digitalization could facilitate connections between physicians and researchers, making it easier to monitor patient needs regarding research.

## Conclusions

This sarcoma PSP has identified priorities for research (e.g. causes of sarcomas, prognosis, etc.) and patient advocacy (e.g. improving the diagnostic process, establishing an international sarcoma registry etc.). It is important to note that many of the research topics ranked high by respondents, are currently unanswered by research. These findings could provide guidance for researchers, assist funding agencies with assessing project relevance and empower patient advocates to represent the needs of patients and carers. We encourage all of these parties to use the results of this survey as a guideline for setting up the research agenda for different patient subgroups.

### Electronic supplementary material

Below is the link to the electronic supplementary material.

Supplementary Material 1

## Data Availability

All data generated or analysed during this study are included in this published article and its supplementary information files.

## Abbreviations

SPAGN: Sarcoma Patient Global Network
PSP: Priority Setting Partnership
PPRN: Patient-Powered Research Network
JLA: James Lind Alliance
BS: Bone sarcomas
STS: Soft tissue sarcomas
GISTs: Gastrointestinal stromal tumors
DF: Desmoid fibromatosis
QoL: Quality of life
HCPs: Healthcare professionals
AYAs: Adolescents and young adults
