# Supplementary material for: Setting the international research agenda for sarcomas with patients and carers: results of phase II of the Sarcoma Patient Advocacy Global Network (SPAGN) priority setting partnership
Source: BMC Cancer. 2024 Aug 6;24:962. doi: 10.1186/s12885-024-12732-6 (PMC11301941; doi:10.1186/s12885-024-12732-6)
Supplement: Supplementary file 1 — Supplementary Material 1 [file 12885_2024_12732_MOESM1_ESM.docx]

# Supplementary

## Supplementary 1 – Questionnaire

**Sarcoma Priority Setting Partnership Survey: part two**

Have you ever had a question about the diagnosis, treatment, survivorship or end-of-life care of sarcoma and not been able to find out the answer? Are there any aspects of sarcoma which you feel should be addressed by research? If so, please take part in this survey.

**Why we need your help?**

We want to know what needs to be improved about the diagnosis, treatment and care of people living with or beyond sarcoma. We want to use your questions to help set priorities for research and patient advocacy. This means: Your experience with this disease will help us understand where research is needed that will make a difference to people’s lives.

**Who can get involved?**

We want to hear from you if you are:

- a person with sarcoma (including gastrointestinal stromal tumors (GIST),desmoid tumors, giant cell tumors and phyllodes)
- a sarcoma survivor or person who once had sarcoma
- a carer for, or family member of, someone with sarcoma
- a bereaved carer or family member of someone who had sarcoma
- a patient advocate

**What are we asking you to do?**

In the first part of the study which ended in early 2022 unanswered questions regarding sarcomas were assessed using open questions. This resulted in a list with topics for future research questions and patient advocacy topics. This questionnaire is developed to help prioritize the unanswered questions and topics. At the end of the survey there is also an opportunity to include any important unanswered questions that you feel are not mentioned in the list. When filling in the questionnaire, please think about your own experiences of sarcoma as a patient, survivor or carer.

By understanding your questions about sarcomas and everything entailed, we can identify the biggest challenges for sarcoma patients and where research and patient advocacy could make the most difference.

*The questionnaire will take 15-20 min to complete. You can participate anonymously if you prefer.*

**What will happen to your question(s)?**

- With the results of this questionnaire a top 5 of research priorities and a top 3 of patient advocacy topics will be identified. At the end of the process, a top 5 list of research questions will be published which will be used to influence future research decisions. Results concerning patient advocacy topics will be communicated to the patient advocacy groups.

**Survey - Section 1 Research priorities**

This table lists the unanswered research questions and research topics that were identified in the first part of the survey. Top 5 of unanswered research questions and topics (fill in the number of the question/topic):

1.

2.

3.

4.

5.

Subjects for research (R)

1. What are causes of sarcoma?
2. Are there ways to prevent sarcoma?
3. Can vaccines be developed to prevent or treat sarcomas?
4. In which way are hereditary aspects involved in the development of sarcoma?
5. More research specific on subtypes of sarcoma (eg GIST, retroperitoneal liposarcoma, angiosarcoma, …) is needed.
6. Which personal characteristics have sarcoma survivors in common (think of psychological, medical and sociodemographic characteristics)?
7. What are the most accurate techniques for the diagnosis of sarcoma (think of imaging modalities, blood tests, whole genome sequencing, etc) and which techniques or strategies could be used to improve the distinction between different subtypes of sarcoma and between benign and malignant tumors?
8. What percentage of people with sarcoma receive the wrong diagnosis in the first instance?
9. What is the risk of taking a biopsy?
10. What is the role of immunotherapy, targeted therapy and combined therapy in the treatment of sarcomas?
11. What is the effect of different treatment modalities on survival and quality of life?
12. More research is needed into novel surgery techniques.
13. What is the effect of different surgical techniques and surgical margins on the outcome for the patient (think of functional outcomes, prognosis, recurrence, etc)?
14. What is the effect of lifestyle (diet, physical activity, etc) on the development of sarcoma?
15. What is the effect of lifestyle on the outcome (e.g. quality of life) during and after treatment?
16. What are the possible treatment methods (e.g. psychotherapy, mindfulness, psychedelics) for disease-related mental suffering (e.g. acceptance, anxiety)?
17. What are the side effects of the different treatment options (targeted therapy, chemotherapy, radiotherapy, surgery, etc.) and how can these side effects be treated?
18. What are the long-term effects of sarcoma treatment on intimacy and fertility?
19. What is the prognosis and the risk of recurrence of sarcoma and which factors have an effect on this?
20. How can follow-up scheme for sarcoma patients be better personalised?
21. How can the re-integration of sarcoma survivors in the society be facilitated (think of work re-integration, social re-integration)?
22. How is end-of-life care organised (in different countries)?
23. What is happening in the terminal phase (development of the disease) and what are the best methods to give best supportive care?
24. What is the role of carers in the final phase of life and how can carers support the patient in taking decisions in the final phase of life?

2.  Is there an important research question that was not listed in the table above? Please explain this question in your own words in the box beneath. *You could think about different aspects such as: diagnosis, treatment, side effects, quality of life, support, follow-up, survival etc* (maximum 20 words)

*<If this topic does not apply to you, please go to the next question>*

**Survey - Section 2 Patient advocacy priorities**

This table lists the patient advocacy topics that were identified in the first phase of the survey. Please fill in a top 3 of patient advocacy topics (fill in the number of the topic):

1.

2.

3.

Subjects for advocacy (A)

1. Improving the diagnostic process of sarcoma through better education and development of tools that can assist general practitioners in recognizing the possibility of a sarcoma.
2. A better classification is needed for benign and malignant tumors. Benign tumors should be included in tumor registries.
3. Analysis of the tumor DNA should be available for all patients.
4. Data sharing should be improved; all relevant data of a patient should be available across medical centers.
5. An international registry with data about sarcoma patients is needed to supply data for research and stimulate international research collaboration.
6. Communication between specialists and patient must be improved to stimulate shared decision-making.
7. A single point of contact must be provided to patients (e.g. case manager, specialized nurse).
8. Information on all tumor subtypes must be available for patients.
9. Sarcoma centers should advise patients on complementary treatments, lifestyle and diet.
10. More attention should be given to quality of life and consequences of treatment (e.g. pain, temporary/permanent effects of surgery, side effects of medication) during the shared-decision making process.
11. Mental support must be available for sarcoma patients.
12. End-of-life scenario should be discussed openly and timely with the patient.
13. Referral of patients to sarcoma expert centers, centralization, networks.
14. The availability to patients of off-label* or compassionate use** medication.
    *The use of a drug that is approved to treat other diseases than your disease
    ** To make an unapproved drug available to treat a disease for which there is no other satisfactory therapy

**Survey – Section 3 About you**

To help us reach a wide range of people, it would be helpful to know a little bit more about you.

1. Are you [please specify one or more]:

- A person who has been diagnosed with and treated for sarcoma
- A carer/partner/relative of a person who has been diagnosed/treated for sarcoma
- A bereaved carer/partner/relative of a person who had sarcoma
- A patient advocate
- Other, please specify_______________________________

1. Are you:

- Male
- Female
- Prefer not to say

1. How old are you?________________________________________________________

- Prefer not to say

1. What is your ethnic origin?

- White
- African American/black
- Asian or Pacific Islander
- Hispanic/Latino
- Multiple/mixed, please specify: __________________________
- Other, please specify: __________________________
- Prefer not to say

1. Which of the following best describes your highest level of education completed?

- No education or primary school
- Secondary school
- College/Diploma
- University/Degree
- Other, please specify __________________________
- Prefer not to say

1. In which country do you live/work? __________________________

- Prefer not to say

1. How long are you (or the person with sarcoma) living with sarcoma?

- <1 year
- ≥ 1 year - < 5 years
- ≥ 5 years - < 10 years
- ≥ 10 years
- Prefer not to say

1. Which of the following statements describes your situation (or the person diagnosed with sarcoma) at the moment? (Please tick all that apply)

- About to start treatment
- Part-way through my treatment
- Finished treatment, date of last treatment _____________________________________________
- Me or the patient with a sarcoma refused (further) treatment tTreatment has been restarted
- No more treatment is needed
- No more treatment is possible
- Under regular follow-up (control checkups)
- Out of follow-up
- Other, please specify _______________________________________________________________
- I don’t know
- Prefer not to say

1. What type of sarcoma were you (or the person with sarcoma) diagnosed with?

- Bone sarcoma
- Soft tissue sarcoma
- GIST (gastrointestinal stromal tumour)
- Desmoid fibromatosis
- Phyllodes
- Giant cell tumor (TCGT)
- I don’t know
- Prefer not to say

1. Which subtype of bone sarcoma or soft tissue sarcoma were you (or the person with sarcoma) diagnosed with? *If you (or the person with sarcoma) are not diagnosed with a bone sarcoma or soft tissue sarcoma, please continue with question 11.*

Bone sarcoma:

- Chondrosarcoma
- Ewing’s sarcoma
- Osteosarcoma
- Chordoma
- Other (please specify)
- I don’t know
- Prefer not to say

Soft tissue sarcoma:

- Sarcoma NOS/Undifferentiated sarcoma
- Well-differentiated/dedifferentiated liposarcoma
- Myxoid liposarcoma
- Leiomyosarcoma
- Dermatofibrosarcoma protuberans
- Solitary fibrous tumour
- Angiosarcoma
- Myxofibrosarcoma
- Synovial sarcoma
- Other (please specify)
- I don’t know
- Prefer not to say

1. What was the location of the sarcoma you (or the person with sarcoma) were diagnosed with?

- Upper limb
- Lower limb
- Head/Neck
- Scalp
- Retroperitoneal
- Heart/vascular
- Lung
- Gynaecologic
- Intra-abdominal
- Torso
- Other:
- I don’t know
- Prefer not to say

1. What was the stage of the sarcoma you (or the person with sarcoma) were diagnosed with?

- Localised
- Metastatic/Disseminated
- I don’t know
- Prefer not to say

1. Did you (or the person you care(d) for) had a recurrence?

- Local (at the site of the primary tumour) or regional (in the lymph system)
- Distant, metastasis
- I don’t know
- Prefer not to say

15. What is the intention of the treatment(s) you (or the person diagnosed with sarcoma) had or you are currently receiving?

- Curative (cure your sarcoma)
- Palliative (help you live longer but not cure the sarcoma)
- Symptom control only
- I don’t know
- Prefer not to say

16. Since first diagnosis, which, if any, of the following treatments have you (or the person diagnosed with sarcoma) had or are you currently receiving? (Please tick all that apply)

- Surgery
- Amputation
- Radiotherapy
- Chemotherapy
- Proton beam therapy
- Targeted therapy
- Immunotherapy
- Hormonal therapy
- Isolated limb perfusion
- Hyperthermia
- I took/am taking part in a clinical trial (testing a new treatment)
- I am not treated (e.g. wait-and-see-policy/watchful waiting)
- Other, please specify _______________________________________________________
- I don’t know
- Prefer not to say

17. Do you (or the person diagnosed with sarcoma) have any other diseases?

- No
- Yes, please specify ___________________________________________________________
- I don’t know
- Prefer not to say

**Next Steps**

Would you like more information about the next stage of the project, where the results of the questionnaire will be published?

- Yes
- No

If 'yes', please supply your contact details below.

**Contact details**

Your contact details will be kept confidential and securely, in accordance with the GDPR.

Name: ___________________________________________________

Email: ___________________________________________________

Postal address: ___________________________________________________

**Consent**

By participating in this survey you are agreeing to allow us to anonymously publish the questions you identify.

Thank you for taking the time to complete this survey. Your participation is important to us – please submit your responses as soon as possible.

*If you have any questions, please contact Dr. Evelyne Roets, PhD student in the Netherlands Cancer Institute: e.roets@nki.nl*

## Supplementary 2 – Research priorities: tumor subgroups

|  | BS (n=83) | STS (n=354) | GIST (n=112) | DF (n=94) |
| --- | --- | --- | --- | --- |
| What are causes of sarcoma? | **51** | **43** | **35** | **45** |
| Are there ways to prevent sarcoma? | **41** | 26 | 24 | **34** |
| Can vaccines be developed to prevent or treat sarcomas? | **34** | 22 | 19 | 18 |
| In which way are hereditary aspects involved in the development of sarcoma? | **46** | 28 | 24 | **27** |
| More research specific on subtypes of sarcoma (eg GIST, retroperitoneal liposarcoma, angiosarcoma, …) is needed. | **35** | **31** | **55** | 12 |
| Which personal characteristics have sarcoma survivors in common (think of psychological, medical and sociodemographic characteristics)? | 30 | 22 | 15 | 13 |
| What are the most accurate techniques for the diagnosis of sarcoma (think of imaging modalities, blood tests, whole genome sequencing, etc) and which techniques or strategies could be used to improve the distinction between different subtypes of sarcoma and between benign and malignant tumors? | 22 | **30** | **28** | 22 |
| What percentage of people with sarcoma receive the wrong diagnosis in the first instance? | 14 | 15 | 9 | 11 |
| What is the risk of taking a biopsy? | 11 | 12 | 12 | 21 |
| What is the role of immunotherapy, targeted therapy and combined therapy in the treatment of sarcomas? | 25 | **33** | **29** | 20 |
| What is the effect of different treatment modalities on survival and quality of life? | 17 | 29 | **29** | **28** |
| More research is needed into novel surgery techniques. | 13 | 9 | 10 | 16 |
| What is the effect of different surgical techniques and surgical margins on the outcome for the patient (think of functional outcomes, prognosis, recurrence, etc)? | 19 | 23 | 14 | 26 |
| What is the effect of lifestyle (diet, physical activity, etc) on the development of sarcoma? | 20 | 19 | 24 | **50** |
| What is the effect of lifestyle on the outcome (e.g. quality of life) during and after treatment? | 7 | 14 | 20 | 18 |
| What are the possible treatment methods (e.g. psychotherapy, mindfulness, psychedelics) for disease-related mental suffering (e.g. acceptance, anxiety)? | 14 | 11 | 12 | 10 |
| What are the side effects of the different treatment options (targeted therapy, chemotherapy, radiotherapy, surgery, etc.) and how can these side effects be treated? | 11 | 15 | 23 | 24 |
| What are the long-term effects of sarcoma treatment on intimacy and fertility? | 4 | 5 | 3 | 16 |
| What is the prognosis and the risk of recurrence of sarcoma and which factors have an effect on this? | 30 | **44** | **42** | **27** |
| How can follow-up scheme for sarcoma patients be better personalised? | 22 | 15 | 9 | 7 |
| How can the re-integration of sarcoma survivors in the society be facilitated (think of work re-integration, social re-integration)? | 6 | 6 | 4 | 2 |
| How is end-of-life care organised (in different countries)? | 1 | 4 | 7 | 2 |
| What is happening in the terminal phase (development of the disease) and what are the best methods to give best supportive care? | 7 | 14 | 17 | 9 |
| What is the role of carers in the final phase of life and how can carers support the patient in taking decisions in the final phase of life? | 1 | 5 | 5 | 4 |

*Numbers in percentages. Top 5 of research priorities in bold. BS=Bone Sarcoma; STS=Soft Tissue Sarcoma; GIST=gastrointestinal stromal tumor; DF=Desmoid Fibromatosis*

## Supplementary 3 – Research priorities: AYAs and older adults.

|  | AYAs (n=105) | >39 years (n=391) |
| --- | --- | --- |
| What are causes of sarcoma? | **44** | **47** |
| Are there ways to prevent sarcoma? | **27** | 28 |
| Can vaccines be developed to prevent or treat sarcomas? | **28** | 20 |
| In which way are hereditary aspects involved in the development of sarcoma? | 22 | **31** |
| More research specific on subtypes of sarcoma (eg GIST, retroperitoneal liposarcoma, angiosarcoma, …) is needed. | 26 | **34** |
| Which personal characteristics have sarcoma survivors in common (think of psychological, medical and sociodemographic characteristics)? | 2**7** | 20 |
| What are the most accurate techniques for the diagnosis of sarcoma (think of imaging modalities, blood tests, whole genome sequencing, etc) and which techniques or strategies could be used to improve the distinction between different subtypes of sarcoma and between benign and malignant tumors? | 21 | 28 |
| What percentage of people with sarcoma receive the wrong diagnosis in the first instance? | 15 | 13 |
| What is the risk of taking a biopsy? | 12 | 15 |
| What is the role of immunotherapy, targeted therapy and combined therapy in the treatment of sarcomas? | 22 | **29** |
| What is the effect of different treatment modalities on survival and quality of life? | 25 | 25 |
| More research is needed into novel surgery techniques. | 15 | 10 |
| What is the effect of different surgical techniques and surgical margins on the outcome for the patient (think of functional outcomes, prognosis, recurrence, etc)? | 26 | 22 |
| What is the effect of lifestyle (diet, physical activity, etc) on the development of sarcoma? | **34** | 24 |
| What is the effect of lifestyle on the outcome (e.g. quality of life) during and after treatment? | 20 | 16 |
| What are the possible treatment methods (e.g. psychotherapy, mindfulness, psychedelics) for disease-related mental suffering (e.g. acceptance, anxiety)? | 10 | 12 |
| What are the side effects of the different treatment options (targeted therapy, chemotherapy, radiotherapy, surgery, etc.) and how can these side effects be treated? | 19 | 17 |
| What are the long-term effects of sarcoma treatment on intimacy and fertility? | 22 | 2 |
| What is the prognosis and the risk of recurrence of sarcoma and which factors have an effect on this? | **35** | **44** |
| How can follow-up scheme for sarcoma patients be better personalised? | 15 | 13 |
| How can the re-integration of sarcoma survivors in the society be facilitated (think of work re-integration, social re-integration)? | 6 | 5 |
| How is end-of-life care organised (in different countries)? | 4 | 4 |
| What is happening in the terminal phase (development of the disease) and what are the best methods to give best supportive care? | 5 | 12 |
| What is the role of carers in the final phase of life and how can carers support the patient in taking decisions in the final phase of life? | 2 | 2 |

*Numbers in percentages. Top 5 of research priorities in bold. AYAs=Adolescents and Young Adults.*

## Supplementary 4 – Research priorities: top 5 respondent countries.

|  | Japan (n=90) | Germany (n=127) | Netherlands (n=94) | UK (80) | Italy (n=74) |
| --- | --- | --- | --- | --- | --- |
| What are causes of sarcoma? | **34** | **39** | **49** | **40** | **53** |
| Are there ways to prevent sarcoma? | 24 | **29** | 22 | 28 | **38** |
| Can vaccines be developed to prevent or treat sarcomas? | **31** | 20 | 3 | 11 | **30** |
| In which way are hereditary aspects involved in the development of sarcoma? | 20 | 28 | **28** | 30 | **35** |
| More research specific on subtypes of sarcoma (eg GIST, retroperitoneal liposarcoma, angiosarcoma, …) is needed. | **56** | 24 | 24 | **36** | 15 |
| Which personal characteristics have sarcoma survivors in common (think of psychological, medical and sociodemographic characteristics)? | 27 | 24 | 21 | 19 | 12 |
| What are the most accurate techniques for the diagnosis of sarcoma (think of imaging modalities, blood tests, whole genome sequencing, etc) and which techniques or strategies could be used to improve the distinction between different subtypes of sarcoma and between benign and malignant tumors? | **27** | **30** | 23 | **31** | 22 |
| What percentage of people with sarcoma receive the wrong diagnosis in the first instance? | 9 | 9 | 20 | **29** | 9 |
| What is the risk of taking a biopsy? | 2 | 17 | 23 | 13 | 11 |
| What is the role of immunotherapy, targeted therapy and combined therapy in the treatment of sarcomas? | 26 | **38** | **26** | **33** | 24 |
| What is the effect of different treatment modalities on survival and quality of life? | **27** | **30** | **35** | **29** | 22 |
| More research is needed into novel surgery techniques. | 16 | 6 | 7 | 10 | 16 |
| What is the effect of different surgical techniques and surgical margins on the outcome for the patient (think of functional outcomes, prognosis, recurrence, etc)? | 17 | 24 | 21 | 21 | 22 |
| What is the effect of lifestyle (diet, physical activity, etc) on the development of sarcoma? | 13 | **30** | **26** | 20 | **38** |
| What is the effect of lifestyle on the outcome (e.g. quality of life) during and after treatment? | 16 | 7 | 23 | 11 | 18 |
| What are the possible treatment methods (e.g. psychotherapy, mindfulness, psychedelics) for disease-related mental suffering (e.g. acceptance, anxiety)? | 11 | 11 | 15 | 13 | 5 |
| What are the side effects of the different treatment options (targeted therapy, chemotherapy, radiotherapy, surgery, etc.) and how can these side effects be treated? | 21 | 15 | 15 | 19 | 19 |
| What are the long-term effects of sarcoma treatment on intimacy and fertility? | 3 | 5 | 4 | 6 | 9 |
| What is the prognosis and the risk of recurrence of sarcoma and which factors have an effect on this? | **48** | **41** | **33** | **45** | **30** |
| How can follow-up scheme for sarcoma patients be better personalised? | 6 | 25 | 14 | 16 | 12 |
| How can the re-integration of sarcoma survivors in the society be facilitated (think of work re-integration, social re-integration)? | 7 | 5 | 5 | 3 | 1 |
| How is end-of-life care organised (in different countries)? | 8 | 6 | 5 | 1 | 4 |
| What is happening in the terminal phase (development of the disease) and what are the best methods to give best supportive care? | 16 | 18 | 17 | 11 | 11 |
| What is the role of carers in the final phase of life and how can carers support the patient in taking decisions in the final phase of life? | 7 | 6 | 6 | 1 | 7 |

*Numbers in percentages. Top 5 of research priorities in bold. UK=United Kingdom.*

## Supplementary 5 - Research priorities: males and females.

|  | Male (n=112) | Female (n=549) |
| --- | --- | --- |
| What are causes of sarcoma? | **54** | **41** |
| Are there ways to prevent sarcoma? | **29** | **29** |
| Can vaccines be developed to prevent or treat sarcomas? | **39** | 18 |
| In which way are hereditary aspects involved in the development of sarcoma? | **31** | **29** |
| More research specific on subtypes of sarcoma (eg GIST, retroperitoneal liposarcoma, angiosarcoma, …) is needed. | **46** | **30** |
| Which personal characteristics have sarcoma survivors in common (think of psychological, medical and sociodemographic characteristics)? | 20 | 20 |
| What are the most accurate techniques for the diagnosis of sarcoma (think of imaging modalities, blood tests, whole genome sequencing, etc) and which techniques or strategies could be used to improve the distinction between different subtypes of sarcoma and between benign and malignant tumors? | 26 | 28 |
| What percentage of people with sarcoma receive the wrong diagnosis in the first instance? | 16 | 14 |
| What is the risk of taking a biopsy? | 13 | 14 |
| What is the role of immunotherapy, targeted therapy and combined therapy in the treatment of sarcomas? | 24 | **31** |
| What is the effect of different treatment modalities on survival and quality of life? | 21 | **29** |
| More research is needed into novel surgery techniques. | 14 | 10 |
| What is the effect of different surgical techniques and surgical margins on the outcome for the patient (think of functional outcomes, prognosis, recurrence, etc)? | 18 | 22 |
| What is the effect of lifestyle (diet, physical activity, etc) on the development of sarcoma? | 22 | 25 |
| What is the effect of lifestyle on the outcome (e.g. quality of life) during and after treatment? | 14 | 15 |
| What are the possible treatment methods (e.g. psychotherapy, mindfulness, psychedelics) for disease-related mental suffering (e.g. acceptance, anxiety)? | 7 | 12 |
| What are the side effects of the different treatment options (targeted therapy, chemotherapy, radiotherapy, surgery, etc.) and how can these side effects be treated? | 17 | 17 |
| What are the long-term effects of sarcoma treatment on intimacy and fertility? | 5 | 6 |
| What is the prognosis and the risk of recurrence of sarcoma and which factors have an effect on this? | **29** | **42** |
| How can follow-up scheme for sarcoma patients be better personalised? | 9 | 15 |
| How can the re-integration of sarcoma survivors in the society be facilitated (think of work re-integration, social re-integration)?] | 5 | 5 |
| How is end-of-life care organised (in different countries)?] | 4 | 4 |
| What is happening in the terminal phase (development of the disease) and what are the best methods to give best supportive care?] | 7 | 14 |
| What is the role of carers in the final phase of life and how can carers support the patient in taking decisions in the final phase of life?] | 4 | 4 |

*Numbers in percentages. Top 5 of research priorities in bold.*

## Supplementary 6 - Research priorities: patients and carers.

|  | Carers (n=170) | Patients* (n=501) |
| --- | --- | --- |
| What are causes of sarcoma? | **35** | **46** |
| Are there ways to prevent sarcoma? | **30** | **28** |
| Can vaccines be developed to prevent or treat sarcomas? | **22** | 22 |
| In which way are hereditary aspects involved in the development of sarcoma? | **31** | **29** |
| More research specific on subtypes of sarcoma (eg GIST, retroperitoneal liposarcoma, angiosarcoma, …) is needed. | **33** | **33** |
| Which personal characteristics have sarcoma survivors in common (think of psychological, medical and sociodemographic characteristics)? | 17 | 22 |
| What are the most accurate techniques for the diagnosis of sarcoma (think of imaging modalities, blood tests, whole genome sequencing, etc) and which techniques or strategies could be used to improve the distinction between different subtypes of sarcoma and between benign and malignant tumors? | **31** | 26 |
| What percentage of people with sarcoma receive the wrong diagnosis in the first instance? | 15 | 14 |
| What is the risk of taking a biopsy? | 13 | 14 |
| What is the role of immunotherapy, targeted therapy and combined therapy in the treatment of sarcomas? | **35** | **28** |
| What is the effect of different treatment modalities on survival and quality of life? | **35** | 25 |
| More research is needed into novel surgery techniques. | 11 | 11 |
| What is the effect of different surgical techniques and surgical margins on the outcome for the patient (think of functional outcomes, prognosis, recurrence, etc)? | 18 | 23 |
| What is the effect of lifestyle (diet, physical activity, etc) on the development of sarcoma? | 21 | 26 |
| What is the effect of lifestyle on the outcome (e.g. quality of life) during and after treatment? | 8 | 17 |
| What are the possible treatment methods (e.g. psychotherapy, mindfulness, psychedelics) for disease-related mental suffering (e.g. acceptance, anxiety)? | 10 | 12 |
| What are the side effects of the different treatment options (targeted therapy, chemotherapy, radiotherapy, surgery, etc.) and how can these side effects be treated? | 16 | 17 |
| What are the long-term effects of sarcoma treatment on intimacy and fertility? | 4 | 7 |
| What is the prognosis and the risk of recurrence of sarcoma and which factors have an effect on this? | **35** | **42** |
| How can follow-up scheme for sarcoma patients be better personalised? | 15 | 13 |
| How can the re-integration of sarcoma survivors in the society be facilitated (think of work re-integration, social re-integration)? | 2 | 6 |
| How is end-of-life care organised (in different countries)? | 5 | 4 |
| What is happening in the terminal phase (development of the disease) and what are the best methods to give best supportive care? | 20 | 10 |
| What is the role of carers in the final phase of life and how can carers support the patient in taking decisions in the final phase of life? | 11 | 2 |

**Including patient advocates*

*Numbers in percentages. Top 5 of research priorities in bold.*

## Supplementary 7 - Research priorities: curative and palliative treatment setting.

|  | Palliative (n=149) | Curative (n=350) |
| --- | --- | --- |
| What are causes of sarcoma? | 36 | 48 |
| Are there ways to prevent sarcoma? | 28 | 32 |
| Can vaccines be developed to prevent or treat sarcomas? | 31 | 22 |
| In which way are hereditary aspects involved in the development of sarcoma? | 19 | 35 |
| More research specific on subtypes of sarcoma (eg GIST, retroperitoneal liposarcoma, angiosarcoma, …) is needed. | 41 | 31 |
| Which personal characteristics have sarcoma survivors in common (think of psychological, medical and sociodemographic characteristics)? | 17 | 22 |
| What are the most accurate techniques for the diagnosis of sarcoma (think of imaging modalities, blood tests, whole genome sequencing, etc) and which techniques or strategies could be used to improve the distinction between different subtypes of sarcoma and between benign and malignant tumors? | 26 | 28 |
| What percentage of people with sarcoma receive the wrong diagnosis in the first instance? | 13 | 13 |
| What is the risk of taking a biopsy? | 13 | 14 |
| What is the role of immunotherapy, targeted therapy and combined therapy in the treatment of sarcomas? | 40 | 28 |
| What is the effect of different treatment modalities on survival and quality of life? | 38 | 21 |
| More research is needed into novel surgery techniques. | 11 | 10 |
| What is the effect of different surgical techniques and surgical margins on the outcome for the patient (think of functional outcomes, prognosis, recurrence, etc)? | 17 | 21 |
| What is the effect of lifestyle (diet, physical activity, etc) on the development of sarcoma? | 21 | 24 |
| What is the effect of lifestyle on the outcome (e.g. quality of life) during and after treatment? | 14 | 14 |
| What are the possible treatment methods (e.g. psychotherapy, mindfulness, psychedelics) for disease-related mental suffering (e.g. acceptance, anxiety)? | 9 | 13 |
| What are the side effects of the different treatment options (targeted therapy, chemotherapy, radiotherapy, surgery, etc.) and how can these side effects be treated? | 15 | 17 |
| What are the long-term effects of sarcoma treatment on intimacy and fertility? | 3 | 6 |
| What is the prognosis and the risk of recurrence of sarcoma and which factors have an effect on this? | 28 | 44 |
| How can follow-up scheme for sarcoma patients be better personalised? | 14 | 14 |
| How can the re-integration of sarcoma survivors in the society be facilitated (think of work re-integration, social re-integration)? | 3 | 6 |
| How is end-of-life care organised (in different countries)? | 9 | 3 |
| What is happening in the terminal phase (development of the disease) and what are the best methods to give best supportive care? | 31 | 7 |
| What is the role of carers in the final phase of life and how can carers support the patient in taking decisions in the final phase of life? | 9 | 3 |

##

*Numbers in percentages. Top 5 of research priorities in bold.*

## Supplementary 8 – Patient advocacy priorities: tumor subgroups.

|  | BS (n=83) | STS (n=354) | GIST (n=112) | DF (n=94) |
| --- | --- | --- | --- | --- |
| Improving the diagnostic process of sarcoma through better education and development of tools that can assist general practitioners in recognizing the possibility of a sarcoma. | **60** | **39** | **33** | **33** |
| A better classification is needed for benign and malignant tumors. Benign tumors should be included in tumor registries. | 24 | 6 | 6 | **40** |
| Analysis of the tumor DNA should be available for all patients. | **46** | **35** | **43** | **32** |
| Data sharing should be improved; all relevant data of a patient should be available across medical centers. | 19 | 23 | 26 | 17 |
| An international registry with data about sarcoma patients is needed to supply data for research and stimulate international research collaboration. | **33** | **43** | 24 | **32** |
| Communication between specialists and patient must be improved to stimulate shared decision-making. | 20 | 18 | 18 | 17 |
| A single point of contact must be provided to patients (e.g. case manager, specialized nurse). | 11 | 17 | 12 | 4 |
| Information on all tumor subtypes must be available for patients. | 0 | 8 | 10 | 3 |
| Sarcoma centers should advise patients on complementary treatments, lifestyle and diet. | 22 | 21 | 19 | 23 |
| More attention should be given to quality of life and consequences of treatment (e.g. pain, temporary/permanent effects of surgery, side effects of medication) during the shared-decision making process. | 22 | 17 | **28** | 29 |
| Mental support must be available for sarcoma patients. | 13 | 17 | 15 | 22 |
| End-of-life scenario should be discussed openly and timely with the patient. | 1 | 7 | 8 | 4 |
| Referral of patients to sarcoma expert centers, centralization, networks. | 20 | 25 | **33** | 17 |
| The availability to patients of off-label or compassionate use medication. | 24 | 24 | 17 | 20 |

*Numbers in percentages. Top 3 patient advocacy topics in bold. BS=Bone Sarcoma; STS=Soft Tissue Sarcoma; GIST=gastrointestinal stromal tumor; DF=Desmoid Fibromatosis*

## Supplementary 9 – Patient advocacy priorities: AYAs and older adults.

|  | AYAs (n=105) | >39 (n=391) |
| --- | --- | --- |
| Improving the diagnostic process of sarcoma through better education and development of tools that can assist general practitioners in recognizing the possibility of a sarcoma. | **45** | **39** |
| A better classification is needed for benign and malignant tumors. Benign tumors should be included in tumor registries. | 19 | 15 |
| Analysis of the tumor DNA should be available for all patients. | **31** | **36** |
| Data sharing should be improved; all relevant data of a patient should be available across medical centers. | 13 | 24 |
| An international registry with data about sarcoma patients is needed to supply data for research and stimulate international research collaboration. | **37** | **35** |
| Communication between specialists and patient must be improved to stimulate shared decision-making. | 16 | 18 |
| A single point of contact must be provided to patients (e.g. case manager, specialized nurse). | 12 | 14 |
| Information on all tumor subtypes must be available for patients. | 3 | 8 |
| Sarcoma centers should advise patients on complementary treatments, lifestyle and diet. | 26 | 21 |
| More attention should be given to quality of life and consequences of treatment (e.g. pain, temporary/permanent effects of surgery, side effects of medication) during the shared-decision making process. | 21 | 24 |
| Mental support must be available for sarcoma patients. | 30 | 15 |
| End-of-life scenario should be discussed openly and timely with the patient. | 4 | 6 |
| Referral of patients to sarcoma expert centers, centralization, networks. | 26 | 24 |
| The availability to patients of off-label or compassionate use medication. | 16 | 19 |

*Numbers in percentages. Top 3 patient advocacy topics in bold. AYAs=Adolescents and Young Adults.*

## Supplementary 10 – Patient advocacy priorities: top 5 respondent countries

|  | Japan (n=90) | Germany (n=127) | Netherlands (94) | UK (80) | Italy (n=74) |
| --- | --- | --- | --- | --- | --- |
| Improving the diagnostic process of sarcoma through better education and development of tools that can assist general practitioners in recognizing the possibility of a sarcoma. | 28 | **45** | **30** | **54** | **36** |
| A better classification is needed for benign and malignant tumors. Benign tumors should be included in tumor registries. | 9 | 5 | 10 | 5 | 27 |
| Analysis of the tumor DNA should be available for all patients. | 34 | **38** | 23 | **38** | **45** |
| Data sharing should be improved; all relevant data of a patient should be available across medical centers. | 29 | 21 | 24 | 24 | 27 |
| An international registry with data about sarcoma patients is needed to supply data for research and stimulate international research collaboration. | 36 | **35** | **36** | **43** | **41** |
| Communication between specialists and patient must be improved to stimulate shared decision-making. | 17 | 20 | 15 | 20 | 22 |
| A single point of contact must be provided to patients (e.g. case manager, specialized nurse). | 9 | 20 | 18 | 20 | 4 |
| Information on all tumor subtypes must be available for patients. | 16 | 6 | 9 | 8 | 0 |
| Sarcoma centers should advise patients on complementary treatments, lifestyle and diet. | 14 | 21 | 21 | 21 | 23 |
| More attention should be given to quality of life and consequences of treatment (e.g. pain, temporary/permanent effects of surgery, side effects of medication) during the shared-decision making process. | 13 | 15 | **34** | 20 | 23 |
| Mental support must be available for sarcoma patients. | 20 | 12 | 17 | 18 | 24 |
| End-of-life scenario should be discussed openly and timely with the patient. | 2 | 6 | 12 | 5 | 5 |
| Referral of patients to sarcoma expert centers, centralization, networks. | 41 | 25 | 18 | 23 | 14 |
| The availability to patients of off-label or compassionate use medication. | 18 | 30 | 27 | 23 | 22 |

*Numbers in percentages. Top 3 patient advocacy topics in bold. UK=United Kingdom.*

## Supplementary 11 – Patient advocacy priorities: males and females.

|  | Male (n=112) | Female (n=549) |
| --- | --- | --- |
| Improving the diagnostic process of sarcoma through better education and development of tools that can assist general practitioners in recognizing the possibility of a sarcoma. | **46** | **38** |
| A better classification is needed for benign and malignant tumors. Benign tumors should be included in tumor registries. | 21 | 13 |
| Analysis of the tumor DNA should be available for all patients. | **42** | **36** |
| Data sharing should be improved; all relevant data of a patient should be available across medical centers. | 22 | 23 |
| An international registry with data about sarcoma patients is needed to supply data for research and stimulate international research collaboration. | **31** | **38** |
| Communication between specialists and patient must be improved to stimulate shared decision-making. | 21 | 19 |
| A single point of contact must be provided to patients (e.g. case manager, specialized nurse). | 14 | 14 |
| Information on all tumor subtypes must be available for patients. | 7 | 7 |
| Sarcoma centers should advise patients on complementary treatments, lifestyle and diet. | 15 | 22 |
| More attention should be given to quality of life and consequences of treatment (e.g. pain, temporary/permanent effects of surgery, side effects of medication) during the shared-decision making process. | 11 | 24 |
| Mental support must be available for sarcoma patients. | 14 | 17 |
| End-of-life scenario should be discussed openly and timely with the patient. | 3 | 7 |
| Referral of patients to sarcoma expert centers, centralization, networks. | 28 | 24 |
| The availability to patients of off-label or compassionate use medication. | 21 | 22 |

*Numbers in percentages. Top 3 patient advocacy topics in bold.*

## Supplementary 12 – Patient advocacy priorities: patients and carers.

|  | Carers (n=170) | Patients* (n=501) |
| --- | --- | --- |
| Improving the diagnostic process of sarcoma through better education and development of tools that can assist general practitioners in recognizing the possibility of a sarcoma. | **36** | **40** |
| A better classification is needed for benign and malignant tumors. Benign tumors should be included in tumor registries. | 6 | 16 |
| Analysis of the tumor DNA should be available for all patients. | **44** | **35** |
| Data sharing should be improved; all relevant data of a patient should be available across medical centers. | 25 | 22 |
| An international registry with data about sarcoma patients is needed to supply data for research and stimulate international research collaboration. | **41** | **35** |
| Communication between specialists and patient must be improved to stimulate shared decision-making. | 21 | 18 |
| A single point of contact must be provided to patients (e.g. case manager, specialized nurse). | 14 | 14 |
| Information on all tumor subtypes must be available for patients. | 5 | 7 |
| Sarcoma centers should advise patients on complementary treatments, lifestyle and diet. | 18 | 22 |
| More attention should be given to quality of life and consequences of treatment (e.g. pain, temporary/permanent effects of surgery, side effects of medication) during the shared-decision making process. | 16 | 24 |
| Mental support must be available for sarcoma patients. | 14 | 18 |
| End-of-life scenario should be discussed openly and timely with the patient. | 9 | 5 |
| Referral of patients to sarcoma expert centers, centralization, networks. | 25 | 24 |
| The availability to patients of off-label or compassionate use medication. | 31 | 19 |

**Including patient advocates*

*Numbers in percentages. Top 3 patient advocacy topics in bold.*

## Supplementary 13 – Patient advocacy priorities: curative and palliative treatment setting.

|  | Palliative (n=149) | Curative (n=350) |
| --- | --- | --- |
| Improving the diagnostic process of sarcoma through better education and development of tools that can assist general practitioners in recognizing the possibility of a sarcoma. | **34** | **43** |
| A better classification is needed for benign and malignant tumors. Benign tumors should be included in tumor registries. | 5 | 16 |
| Analysis of the tumor DNA should be available for all patients. | **45** | **35** |
| Data sharing should be improved; all relevant data of a patient should be available across medical centers. | 21 | 22 |
| An international registry with data about sarcoma patients is needed to supply data for research and stimulate international research collaboration. | **42** | **35** |
| Communication between specialists and patient must be improved to stimulate shared decision-making. | 17 | 18 |
| A single point of contact must be provided to patients (e.g. case manager, specialized nurse). | 9 | 16 |
| Information on all tumor subtypes must be available for patients. | 7 | 6 |
| Sarcoma centers should advise patients on complementary treatments, lifestyle and diet. | 20 | 21 |
| More attention should be given to quality of life and consequences of treatment (e.g. pain, temporary/permanent effects of surgery, side effects of medication) during the shared-decision making process. | 21 | 23 |
| Mental support must be available for sarcoma patients. | 13 | 18 |
| End-of-life scenario should be discussed openly and timely with the patient. | 15 | 3 |
| Referral of patients to sarcoma expert centers, centralization, networks. | 21 | 27 |
| The availability to patients of off-label or compassionate use medication. | 30 | 18 |

*Numbers in percentages. Top 3 patient advocacy topics in bold.*
